# A case of matrix-producing carcinoma of the breast

**DOI:** 10.1186/1477-7819-6-60

**Published:** 2008-06-17

**Authors:** Toshiyuki Hirose, Junko Honda, Yoshimi Bando, Mitsunori Sasa, Yukiko Hirose, Taeko Nagao, Akira Tangoku

**Affiliations:** 1 Department of Surgery, National Higashi Tokushima Hospital, 1-1, Ohmukai-kita, Ootera, Itano, Tokushima 779-0193, Japan; 2 Department of Oncological and Regenerative Surgery, Institute of Health Biosciences, The University of Tokushima, 3-18-15, Kuramoto-Cho, Tokushima 770-8509, Japan; 3 Department of Molecular and Environmental Pathology, Institute of Health Biosciences, The University of Tokushima Graduate School, 3-18-15, Kuramoto-Cho, Tokushima 770-8509, Japan; 4 Department of Surgery, Tokushima Breast Care Clinic, 4-7-7, Nakashimada-Cho, Tokushima 770-0052, Japan

## Abstract

**Background:**

Matrix-producing carcinoma (MPC) of the breast is one variant type of metaplastic carcinoma. The cellular origin of MPC remains unclear. It has been suggested the tumor cells in MPC have the combined characteristics of both epithelial cells and mesenchymal cells. Several reports suggested that the tumor cells in MPC might originate from the myoepithelial cells, but others suggested the origin was basal-like cells.

**Case presentation:**

The patient was a 42-year-old Japanese female. A tumor of about 2 cm in diameter was noted in the right breast. CT revealed the circumference of the tumor to have a ring-like structure, and fine needle aspiration cytology indicated suspicion for malignancy. Breast-conserving surgery was performed. Histopathological studies showed carcinoma cells, having cuboidal to oval-shaped nucleus, were proliferating in cord-like and sheet-like structures in the periphery. In the central areas of the tumor, myxoedematous area was observed with cartilaginous matrix and necrosis. The diagnosis was a matrix-producing carcinoma. Immunohistochemical findings showed the tumor cells had the characteristics of both epithelial cells and mesenchymal cells, while being negative for estrogen receptor, progesterone receptor, Her2, myoepithelial cell markers and basal cell markers.

**Conclusion:**

The findings for our present patient and many of the other MPC patients reported in the published literature indicate that this breast cancer has the properties of both epithelial cells and mesenchymal cells. In addition, there is a possibility that matrix-producing tumor cells of our present patient may have a feature of undifferentiated cells.

## Background

Matrix-producing carcinoma (MPC) is a rare and characteristic variant type of metaplastic carcinoma of the breast. In 1989, Wargotz *et al*., proposed defining MPC as overt carcinoma with direct transition to a cartilaginous and/or osseous stromal matrix cells, with no spindle cells between those two elements [1]. In Japan, MPC of the breast was added to the General Rules for Clinical and Pathological Recording of breast Cancer (16^th ^Edition)(The Japanese Breast Cancer Society) as a special form of carcinoma [2]. It was reported that MPC cells had the combined characteristics of both epithelial cells and mesenchymal cells [3-5]. Wargotz *et al*., suggested the tumor cells of MPC might be of epithelial-myoepithelial derivation depending on immunohistochemical analysis and electron microscopic results [1]. In addition, mouse model using *Brca1 *deficiency has suggested an important role for BRCA1 in basal-like breast carcinoma with metaplastic elements [6]. There is thus no consensus in histogenesis of MPC. We report our findings for a Japanese woman with MPC of the breast and discuss them together with the other 26 cases of MPC of the breast that have been reported in Japan to date.

## Case presentation

The patient was a 42-year-old Japanese female with a chief complaint of a lump in her right breast. Her personal and family histories contained nothing of special note.

**Figure 1:**
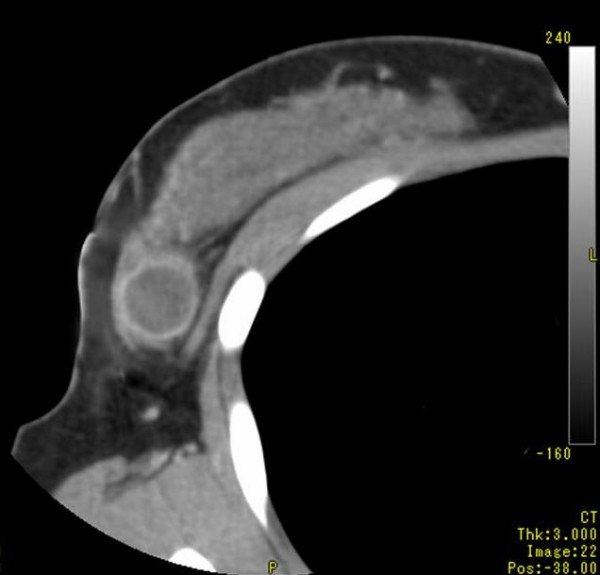
Computed tomography scan imaging (CT) of the tumor. Contrast-enhanced CT revealed, in the right lateral-upper quadrant, an irregularly shaped, 2,5 cm tumor showing peripheral ring-shaped contrast enhancement.

Examination revealed a hard tumor with a clear boundary and a diameter of about 2 cm in the lateral-upper quadrant of the right breast. The axillary and supraclavicular lymph nodes could not be palpated. Mammography revealed focal asymmetric density in the right lateral-upper quadrant, accompanied by amorphous microcalcification. Breast echography indicated a tumor with dimensions of 2.3 × 1.8 × 1.5 cm and a slightly indistinct boundary, and the internal portion was heterogeneous and included a hyperechoic region. Contrast-enhanced CT revealed, in the right lateral-upper quadrant, an irregularly shaped, 2.5 cm tumor showing peripheral ring-shaped contrast enhancement (Fig. 1). There was no evidence of lymph node metastasis or clear distant metastasis. Aspiration cytology showed many tumor cells, having cuboidal to oval-shaped nucleus, were observed in the myxoedematous background containing much necrotic material, but without any sarcomatous spindle cells. The myxoedematous matrix in the background stained pale gray with Papanicolau stain. The tumor cells were isolated, in loosely cohesive groups and in short chains. The nuclear to cytoplasmic ratio was high with coarsely granular chromatin. The diagnosis was suspicious for malignancy. No particular abnormalities were noted on laboratory data, and tumor markers were all within their normal ranges: 1.6 ng/ml for CEA and 19 ng/ml for CA 15-3. Right breast cancer was suspected on the basis of the above findings, and lumpectomy was performed.

### Histopathological findings

**Figure 2:**
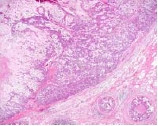
Low-magnification view of the tumor. The central myxoedematous area contained an extensive area of necrosis at its center (HE).

**Figure 3:**
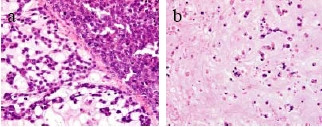
High-magnification view of the peripheral epithelial area (a) and the central area (b) of the tumor. The peripheral epithelial area consisted of cord-like and sheet-like structures of proliferating carcinoma cells having a cuboidal to oval-shaped nucleus. In the central areas of the tumor, sparse distribution of oval tumor cells was observed (HE).

**Figure 4:**
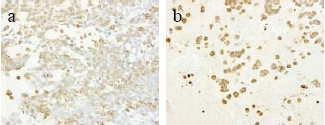
Immunohistochemical staining for vimentin of the peripheral epithelial area (a) and the central myxoedematous area (b). The tumor cells of the both area stained positively for vimentin.

**Figure 5:**
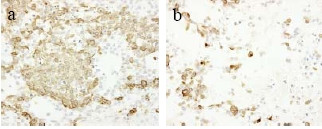
Immunohistochemical staining for cytokeratin AE1/AE3 of the peripheral epithelial area (a) and the central myxoedematous area (b). The tumor cells of the both area stained positively for cytokeratin AE1/AE3.

The tumor consisted of a peripheral epithelial area and a central myxoedematous area. The peripheral epithelial area consisted of cord-like and sheet-like structures of proliferating carcinoma cells having a cuboidal or oval-shaped nucleus. The central myxoedematous and chondroid-looking matrix contained an extensive area of necrosis, but no definite chondrocytes or osseous differentiation (Figs. 2, 3). Ductal carcinoma *in situ *(DCIS) with comedo necrosis was found adjacent to the main tumor. Immunohistochemically, the tumor cells in both the peripheral epithelial area and the central myxoedematous area were negative for estrogen receptor (ER), progesterone receptor (PgR) and Her2. In addition, the tumor cells of both areas stained positively for both vimentin and S-100 protein (Fig. 4), and they also showed partial positive staining for each of cytokeratin AE1/AE3, CK7, CK8 and CK19 (Fig. 5). Conversely, the tumor cells of both areas stained negatively for each of α-smooth muscle antigen (α-SMA), p63 and glial fibrillary acidic protein (GFAP), which were myoepithelial cell markers. The tumor cells were also negative for each of the basal markers, i.e., CK5/6, CK14, CK17, and epidermal growth factor receptor (EGFR). Appropriate human breast cancers known to express ER, PgR and Her2 were included in each slide run. The luminal cells of non-tumorous adjacent ducts were positive with ER and PgR as internal controls. The myoepithelial cells of adjacent non-tumorous ducts and acini were also positive with α-SMA, p63, CK5/6, CK14 and CK17. Detailed information of the immunohistochemistry procedures and the antibodies used was listed in the Table 1. In special staining, the tumor stroma stained positive with alcian blue (pH2.5), which was partially eliminated by digestion with hyaluronidase.

**Table 1:** Characteristics of the overt carcinoma cells in matrix-producing carcinoma of the breast in Japan

	ER	PgR	Her2	EMA	AE1/AE3	Desmin	α-SMA	GFAP	p63	S-100	Vimentin
(+)	0	1	0	16	20	0	4	1	0	15	17
(-)	23	22	14	0	1	11	15	0	1	0	3

ER: Estrogen receptor

PgR: Progesterone receptor

Her2: Human epidermal growth factor 2

EMA: epithelial membrane antigen

GFAP: glial fibrillay acidic protein

SMA: α-smooth muscle actin

### Clinical course

Axillary lymph node dissection was performed on the basis of a definitive diagnosis of MPC of the breast, but there were no findings of metastasis. The remaining breast tissue was irradiated with a total of 50 Gray. Postoperative adjuvant chemotherapy was recommended, but the patient refused this approach and it was thus not administered. Ten months after the axillary surgery, multiple metastases and liver metastasis were diagnosed, and the patient died 8 months after recurrence of the disease.

## Discussion

Epithelial-mesenchymal transition has been reported to be an etiological factor in metaplastic carcinoma [7]. The overt carcinoma cells of almost all of the MPC breast cancer cases reported in Japan were positive for both epithelial cell markers and mesenchymal cell markers (Table 2). Electron microscope findings and the results of immunohistochemical studies were reported to indicate that MPC is of myoepithelial cell origin [1,8]. On the other hand, Okuyama et al. examined specimens from 8 patients and reported that the overt carcinoma cells of all of those cases were negative for myoepithelial cell markers [3]. Moreover, Only 4 of the 27 patients in Japan was positive for myoepithelial cell markers. In the patient we have described, as well, the overt carcinoma cells were positive for vimentin, S-100 protein and cytokeratins (AE1/AE3, CK7, CK8 and CK19). They showed negative staining for α-SMA, p63, CK5/6 and GFAP, which are myoepithelial cell markers. p63 has been reported to be useful as diagnostic marker for metaplastic carcinoma [9,10]. It was reported that the carcinoma cells with spindle and/or squamous differentiation in metaplastic carcinoma showed positive staining for p63. The malignant component with no squamous or sarcomatous differentiation in MPC of our patient might cause negative staining for p63.

**Table 2:** Sources, dilution and pretreatment of antibodies used

**Antibody**	**Clone**	**Manufacturer**	**Dilution**	**Pretreatment**
ER	1D5	DakoCytomation, USA	1:50	boiling (pH9.0, 40 min)
PgR	PgR636	DakoCytomation, USA	1:800	boiling (pH9.0, 40 min)
HER2	DakoCytomation, USA	Prediluted (Hercep test)	boiling (pH6.0, 40 min)	
CK5/6	D5/16B4	DakoCytomation, Denmark	1:100	autoclave (pH6.0, 10 min)
CK14	LL002	NeoMarkers, USA	1:100	autoclave (pH6.0, 10 min)
CK17	E3	DakoCytomation, Denmark	1:40	autoclave (pH6.0, 10 min)
EGFR	2-18C9	DakoCytomation, USA	Prediluted (pharmDX kit)	proteinase K (room temperature, 5 min)
AE1/AE3	AE/AE3	DakoCytomation, Denmark	1:50	pronase (37 C, 15 min)
CK7	OV-TL12/30	DakoCytomation, Denmark	1:50	pronase (37 C, 15 min)
CK8	35βH11	DakoCytomation, USA	1:50	pronase (37 C, 15 min)
CK19	RCK108	DakoCytomation, Denmark	1:50	autoclave (pH6.0, 10 min)
α-SMA	1A4	DakoCytomation, Denmark	1:100	
P63	4A4	DakoCytomation, Denmark	1:50	autoclave (pH6.0, 10 min)
GFAP	6F2	DakoCytomation, Denmark	1:100	

Our patient's MPC exhibited the same cell marker profile as that reported by Okuyama *et al*., showing the properties of both epithelial cells and mesenchymal cells. It was reported that the results of immunohistochemistry differed between the peripheral epithelial area and the central myxoedematous area. In the central myxoedematous area, which can be thought to be causing metaplasia, down-regulation of epithelial markers and up-regulation of mesenchymal markers were observed [1,4,5,11]. On the contrary, for our patient, the peripheral epithelial area and the central my edematous area showed no differences in their staining profiles. Recent molecular studies have shown the monoclonal origin of carcinosarcoma of the breast, as the carcinomatous and sarcomatous elements share common genetic alterations [12,13]. These observations support the hypothesis that the matrix-producing carcinoma may be derived from a single totipotent stem cell.

Our patient had triple-negative breast cancer with regard to ER, PgR and Her2. In addition, it is interesting that almost all of the reported Japanese cases of MPC of the breast were triple-negative. It is said that most cases of metaplastic carcinoma are also triple-negative breast cancer [14,15], and this is important in terms of elucidating the etiology of the metaplastic change. Ninomiya *et al*., reported that one of their two cases of MPC of the breast was the basal phenotype [5]. McCarthy *et al*., generated a conditional mouse model of *BRCA1 *deficiency. Mammary tumors that developed in these mice had basal and metaplastic characteristics in the form of spindle cell and squamous cell differentiation. Most of the tumors were negative for ER, PgR and Her2 [6]. Additionally, a recent report has shown that epithelial mesenchymal transition-like changes occurred preferentially in the basal-like subtype of breast carcinomas [16]. Furthermore, subpopulations of cancer cells with stem cell properties are especially frequent within basal-like breast cell lines [17]. Stem cell-like breast cell lines are also able to undergo epithelial mesenchymal transition [18]. These data suggest that basal-like cancer cells may undergo epithelial mesenchymal transition with intrinsic phenotype of cancer stem cells. Although most cases of triple-negative breast cancer have the basal-like phenotype [6], MPC of our patient had lack of any markers for myoepithelial cell type and basal-like cell type. *BRCA1 *has been shown to play an important role in mammary differentiation and the loss of *BRCA1 *function resulted in the accumulation of cells expressing the stem/progenitor marker ALDH-1 [19]. Although the *BRCA1 *status of our patient has not been identified, it was suggested the possibility that the tumor cells of our MPC might be blocked differentiation with expansion of undifferentiated cell compartment.

Okuyama *et al*., reported that the incidence of MPC of the breast was 0.05% in Japan [4]. Our search of the main Japanese medical journals found a total of 27 cases of MPC of the breast reported in Japan to date, including our present patient [3-5,11]. Imaging diagnosis by contrast-enhanced CT and contrast-enhanced MRI have revealed that this disease is characterized by a ring structure in its periphery. For that reason, it was concluded that it is necessary to consider the possibility of MPC of the breast when such image findings are obtained [3]. In our present patient, as well, contrast-enhanced CT revealed an irregularly shaped, 2.5 cm tumor showing peripheral ring-shaped contrast enhancement.

Most MPC of the breast are triple-negative, and postoperative adjuvant chemotherapy is often administered [13]. However, some studies have shown this therapy to have been ineffective, and further research on this issue is warranted.

The prognosis of MPC of the breast is said to be better than that of other carcinomas that are accompanied by osteocartilaginous metaplasia [20,21]. Wargotz *et al*., reported a 5-year survival rate of 68% for MPC of the breast [1], but the number of reported cases has been small and the prognosis thus remains unclear. Our patient refused postoperative adjuvant chemotherapy, and distant metastasis was detected at 10 months after the partial mastectomy. In the future it will be necessary to study a larger number of patients with MPC of the breast and further elucidate the clinicopathological characteristics of this malignancy.

## Conclusion

There have been reports that MPC of the breast is of myoepithelial cell origin or basal cell origin. However, the findings for our present patient suggested that MPC might be produced as a result of the undifferentiation process.

## List of abbreviations

MPC: Matrix producing carcinoma; ER: Estrogen receptor; PgR: Progesterone receptor; Her2: Hercep test; α-SMA: α-smooth muscle antigen; GEAP: Glial fibrillary acidic protein; EGFR: Epidermal growth factor receptor

## Competing interests

The authors declare that they have no competing interests.

## Authors' contributions

HT, MS and NT took part in the care of the patient, YB examined surgical specimen and took photomicrographs of the slides, JH and MS initiated and co-wrote the paper with TH and AT. All authors read approved the final manuscript.
